# Cervical leukocytes and spontaneous preterm birth

**DOI:** 10.1016/j.jri.2015.11.002

**Published:** 2016-02

**Authors:** Patricia J. Hunter, Sairah Sheikh, Anna L. David, Donald M. Peebles, Nigel Klein

**Affiliations:** a UCL Institute of Child Health, 30 Guilford Street, London WC1N 1EH, UK; b UCL Institute for Women’s Health, 86-96 Chenies Mews, London WC1E 6HX, UK; c University of Oxford Nuffield Department of Obstetrics and Gynaecology, John Radcliffe Hospital, Headington, Oxford OX3 9DU, UK

**Keywords:** SPTB, spontaneous preterm birth, LMC, late miscarriage, PMN, polymorphonuclear cells, Spontaneous preterm birth, Flow cytometry, Leukocyte, Macrophage, Polymorphonuclear cell

## Abstract

The objective was to characterise cervical leukocyte populations and inflammatory mediators associated with term and recurrent spontaneous preterm birth (SPTB) in pregnant women with a history of SPTB. A prospective observational study was undertaken on 120 women with a history of SPTB. A cytobrush was used to sample cells from the cervix at 12–25 weeks’ gestation. Cells were enumerated and characterised by flow cytometry. Cytokines and chemokines were also measured. Participants were then grouped according to delivery at term (>36 + 6 weeks), late SPTB (34–36 + 6 weeks) or early SPTB (<34 weeks). Differences in leukocyte sub-populations, cytokine and chemokine levels were compared with outcome. Cervical leukocytes comprised up to 60% of the host-derived cells. Most of these (90–100%) were polymorphonuclear cells (PMN). Most of the remaining cells were mucosal macrophages expressing CD68 and CD103 in addition to markers shared with blood-borne monocytes. Failure to detect cervical macrophages in at least 250,000 cervical epithelial cells was a feature of women who experienced early SPTB (6 out of 6 cases, 95% CI 61–100%) compared with 34% (30 out of 88 cases, 95% CI 25–43%, *P *< 0.001) of women delivering after 34 weeks. CCL2 (MCP-1) was also low in SPTB before 34 weeks and levels above 75 ng/g and/or the presence of macrophages increased the specificity for birth after 34 weeks from 66% to 82% (55 out of 67 cases, 95% CI 73–91%). Absence of cervical macrophages and low CCL2 may be features of pregnancies at risk of early SPTB.

## Highlights

• Hypothesis that defects in cervical immunity might be a feature of spontaneous preterm birth (SPTB).
• A prospective observational study of 120 pregnancies at risk of SPTB because of the mother’s history.
• Cervical leukocytes, cytokines and chemokines were sampled at 12–25 weeks using a cytobrush.
• Early SPTB (<34 weeks) lacked cervical macrophages and had low CCL2 (<75 ng/g total protein).

## 1 Introduction

Preterm birth (birth before 37 weeks’ gestation) affects 5–13% of births in the developed world and the incidence is increasing (Chang et al., 2013). While factors such as an increase in multiple pregnancies and iatrogenic preterm birth contribute to the overall increase, most spontaneous preterm births (SPTB) remain unexplained. A better understanding of the pathways leading to SPTB and the use of this knowledge to identify women who would most benefit from treatment strategies to prolong gestation would contribute to the alleviation of this societal and healthcare burden.

We and others have compared placentas and fetal membranes delivered at term and preterm for the presence of bacteria using highly sensitive methods (Doyle et al., 2014, Jones et al., 2009, Kim et al., 2009). Although bacteria, most commonly *Lactobacilli* spp., can be found in the placenta and fetal membranes of term deliveries, spontaneous preterm deliveries are distinguished by the much higher frequency of colonisation and by the nature of the species identified. Bacteria can infect and sometimes cross the placenta via the maternal circulation, but in most cases bacteria are thought to access the foetus by ascending the cervical canal, causing chorioamnionitis (infiltration of the membranes by polymorphonuclear cells [PMN]) and colonising the amniotic fluid (DiGiulio et al., 2008, Goldenberg et al., 2000, Kim et al., 2009). As the infection spreads, the normally anti-inflammatory uterine environment may undergo a transformation and begin to respond to bacterial invasion with inflammation; cytokine and chemokine production and the infiltration of both fetal and maternal PMN. Levels of IL-8, IL-6, IL1β, TNFα and the CC type chemokines in maternal serum and cervical secretions increase concomitantly with both term and preterm cervical ripening (Dubicke et al., 2010, Törnblom et al., 2005), pre-labour membrane rupture (Holst et al., 2007, Luo et al., 2000) and contractions (Arntzen et al., 1997, Hebisch et al., 2004, Shobokshi and Shaarawy, 2002, Tency, 2014).

Ascending infection is normally prevented by formidable defence mechanisms that work together to create an environment within the cervical canal that is hostile to bacteria. Anti-microbial peptides (defensins), secreted antibodies and the thick, negatively charged mucus contribute to form an effective antimicrobial shield (Cole, 2006). During pregnancy the mucus plug not only provides a physical barrier, but also retains antibodies and defensins in its matrix, thus enhancing maternal protection from infection (Hein et al., 2002).

In view of this array of antimicrobial defences, it is unclear why in some women bacteria still gain access to the feto-placental unit. To understand why this occurs, we have characterised the leukocyte populations found at the distal cervix and endocervical canal and measured the cytokines and chemokines that mediate leukocyte recruitment. We have adapted a previously described method of sampling the cervix (Prakash et al., 2001, Whitworth et al., 2007) using a cytobrush and extensively characterised the leukocyte populations using flow cytometry. Our hypothesis is that some insufficiency of leukocyte recruitment to the cervix may be associated with a recurrence of SPTB in women with a history of SPTB.

## 2 Methods

### 2.1 Patients and samples

Pregnant women were recruited over a period of 18 months from a specialist antenatal prematurity clinic at University College London Hospital at their first presentation to the clinic. The study was approved by the Harrow Local Research Ethics Committee (09/H0714/66) and has a portfolio (13818) in the NIHR UK Clinical Research Network. Women with a history of at least one spontaneous late miscarriage (spontaneous labour with delivery between 16 + 0 and 23 + 6 weeks’ gestation) or live birth before 36 + 6 weeks’ gestation following spontaneous labour or preterm pre-labour rupture of membranes (PPROM) were invited to participate. Prospective exclusion criteria included previous surgery to the cervix, uterine malformation, multiple pregnancy or a history of iatrogenic preterm delivery. Women were also excluded if they had already received antibiotics, progesterone or cervical cerclage in their current pregnancy. Women who received antibiotics, progesterone or ultrasound-indicated or elective cervical cerclage placement subsequent to study entry were not excluded. Treatments were noted and reported in Table 1. Written informed consent was obtained for each participant.

At presentation to the Preterm Birth Clinic, a history was taken and a urine sample was screened for asymptomatic bacteriuria. Women meeting the criteria for study entry were given information on the study and invited to participate. A sterile speculum examination was performed by a provider with training in Genitourinary Medicine and high vaginal swabs were taken and tested for bacterial vaginosis (growth of anaerobic bacteria), *Trichomonas vaginalis*, gonococcus, chlamydia, group B *Streptococcus* and candidiasis. For women who had consented to participate in the study, a cervical cytobrush (Cellpath, Powys, UK) was then gently rotated 360° in the cervical canal. The brush was placed in a 15-mL tube containing 2 mL of cold phosphate buffered saline (PBS) supplemented with 1 mmol glutamine and pen/strep and kept at 4 °C for up to 4 h while awaiting preparation for flow cytometry.

Fortnightly cervical length measurements were carried out using transvaginal ultrasound on all women attending the Preterm Birth Clinic until 22 weeks’ gestation, when vaginal fetal fibronectin was measured. Data on cervical length and fetal fibronectin for women in the study are reported in Table 1.

### 2.2 Sample processing

Samples were prepared according to Prakash et al. (2001). Briefly, the 15-mL tube containing the cytobrush was vortexed for 1 min, then centrifuged at 200 × *g* for 10 min at 4 °C. The supernatant was aliquoted and saved at −80 °C. The cell pellet was resuspended in 3 mL PBS containing 1% fetal calf serum and passed through a 70-μm filter. Cells in suspension were observed and counted using microscopy. The cells were then centrifuged again at 200 × *g* and resuspended at 10^7^/mL. 30 μL or 300,000 cells were subjected to analysis with groups of antibodies against surface proteins using flow cytometry.

### 2.3 Analysis by flow cytometry

The phenotype of cells collected on the cervical brushes was determined using flow cytometry. Squamous epithelial cells and dead leukocytes absorbed 4′,6-diamino-2-phenylindole (DAPI). Cells that excluded DAPI were examined using directly labelled monoclonal antibodies against the following human proteins: CD45-FITC and CD16-PerCPCy5.5 from BD Biosciences (Franklin Lakes, NJ, USA), CD163-PE, CD11b-PE, CD20-PE, CD14-PerCPCy5.5, HLA-DR-PECy7, CD19-PECy7, CD27-PECy7, CCR7-PECy7, CD11b-APC (activation epitope CBRM1/5) and CD16-APC from eBioscience (San Diego, CA, USA); CD235a-PE, CD68-PE, CD66b-PE, CD103-PE, CD11c-PE, CX3CR1-PE, CCR2-PerCPCy5.5, CD3-PECy7, CCR1-APC, CD4-APC, CD33-APC and CD64-APC from Biolegend (San Diego, CA, USA). Flow cytometric data were collected on an LSRII (BD Biosciences). A minimum of 300,000 events were collected for each group of antibodies so that even a leukocyte population consisting of 0.01% of the total host-derived cells could be reliably detected. Data were analysed using FlowJo 9.6.4 (TreeStar, Ashland, OR, USA).

### 2.4 Protein quantification

Total protein content of cervical brush supernatants was determined by mixing 25 μL with 225 μL of Bradford Reagent (Sigma) in duplicate and comparing the OD at 610 nm with a set of standards containing a known quantity of bovine serum albumin (Sigma). IL-8 was detected using the Ready Set Go 2nd generation ELISA kit from eBioscience. IL-1β, IL-6, TNFα, IFNγ, IL-10, CCL2, CCL3 and CCL4 were detected as a multiplex using the V-plex system from Mesoscale Discovery (MSD, Rockville, MD, USA).

### 2.5 Histological chorioamnionitis

Routine examination for histological chorioamnionitis was performed on the extraembryonic material from all births that occurred up to 34 weeks.

### 2.6 Statistics

Statistical analyses were performed using Statistical Package for the Social Sciences version 18 (SPSS, Chicago, IL, USA) or GraphPad Prism 5 (San Diego, CA, USA)**.** Confidence intervals for sample sizes <15 were calculated using the method of Altman et al. (2000) A Mann–Whitney *U* test was performed to calculate statistical significance between groups. Samples that had no detectable cytokine or chemokine were given the value of the detection limit (12.5 pg/mL for CCL3 and 3 pg/mL for CCL4).

## 3 Results

### 3.1 Clinical characteristics of the cohort

Samples were collected from 120 participants between 12 and 25 weeks’ gestation, but most (85%) were obtained before 18 weeks. Samples contaminated with blood were excluded (*n* = 14, 12%), as were those with fewer than 250,000 cells (*n* = 7, 6%). There was good agreement between the detection of blood by visual inspection and the presence of CD235a-expressing cells detected by flow cytometry. Blood contamination was more common at the beginning of the study and reduced with experience in sampling. Insufficient cell numbers were an unintended consequence of avoiding cervical bleeding. In total, 99 samples were analysed by flow cytometry, but 5 women were subsequently excluded, leaving 94 available for analysis (2 women had iatrogenic preterm births, one woman had an early miscarriage, one woman withdrew from the study and one woman was lost to follow-up). Table 1 shows the clinical characteristics of the participants at the time of sample donation. Among the three outcome categories (≥37 weeks, 34–36 + 6 weeks and <34 weeks) there were no significant differences in the mean age, BMI, smoking during pregnancy, ethnic background or proportion of samples donated before 20 weeks. The proportions of participants with a history of at least one live birth before 34 weeks or who received progesterone, elective cerclage, ultrasound-indicated cerclage owing to a cervix of <25 mm and/or had >50 ng/mL cervico-vaginal fetal fibronectin at 22–24 weeks were increased in the groups of women who had a preterm birth (Table 1), as would be expected using current risk stratification algorithms. Clinical characteristics of the participants whose samples were excluded because of blood contamination or insufficient cell numbers are given in Supplementary Tables 1 and 2.

### 3.2 Surface protein expression by cervical leukocytes

**Fig. 1:**
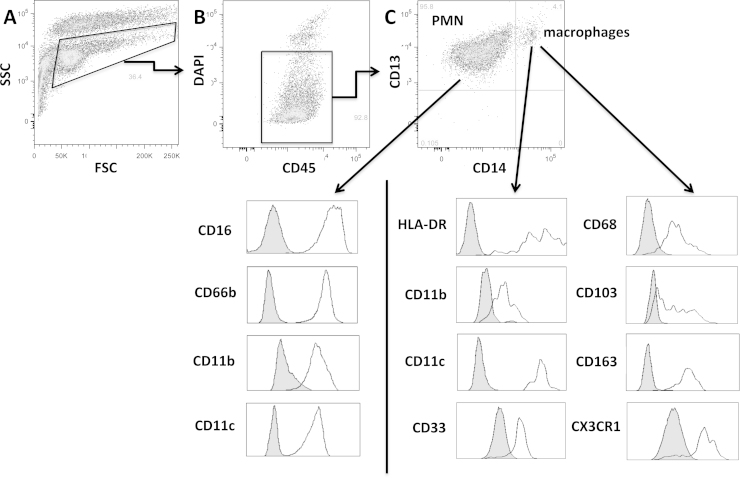
Human cervical leukocytes obtained from cytobrushes consist of two subsets of phagocytic cells, as detected using flow cytometry. Cells in suspension were stained with the antibodies against the indicated surface proteins. (A) Leukocytes could be distinguished from epithelial cells based on size (FSC) and granularity (SSC). (B) Selected events in (A) express CD45. (C) Live cells (those that exclude 4′,6-diamino-2-phenylindole [DAPI]) selected in (B) are either PMN (CD13+, CD16+, CD66b+) or macrophages (CD13+, CD14+, CD68+, HLA-DR+, CD33+, CD163+, CX3CR1+, CD103 variable). Both cell types express CD11c and the activated form of CD11b. Grey histograms represent isotype controls.

**Fig. 2:**
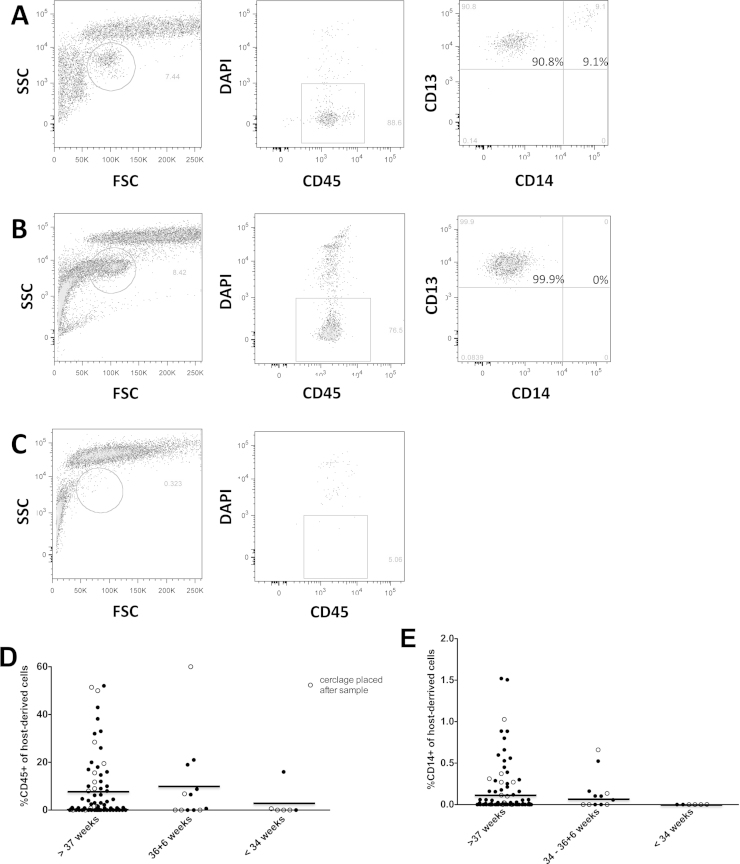
Relationship between cervical cellular phenotype and preterm birth. (A–C) Examples of three different cellular phenotypes from cervical brushes. (A) Leukocyte population consisting of 9% macrophages (CD13+, CD14+) and 91% PMN (CD13+, CD14−). (B) Leukocyte population consisting solely of PMN. (C) No leukocytes detected. (D) Dot plot showing proportion of host-derived cells expressing CD45 from participants who delivered at term (*n* = 76), late preterm (34–36 + 6 weeks; *n* = 12) and early preterm (22–33 + 6 weeks; *n* = 6). (E) Dot plot showing the proportion of host-derived cells expressing CD14 from the same participants as in (D). Open circles indicate participants who received cerclage after sample donation. Horizontal lines indicate means.

In all samples, squamous epithelial cells could be identified by flow cytometry as those with variable sizes and high granularity (Fig. 1A). In most of the samples, a population of CD45+ cells (leukocytes) was also detected (Fig. 1, Fig. 2A and 2B). When present, the majority of leukocytes (90–100%) were polymorphonuclear (PMN), identified by the surface expression of CD13, CD16, CD66b, CD11c and the activated form of CD11b (Fig. 1C). The remaining population largely consisted of cells of the monocyte lineage, uniformly expressing HLA-DR, CD11b, CD11c, CD13, CD14, CD33, CD68, CD163CX3CR1 and a range of CD103 (integrin αE; Fig. 1C). Consistently high expression of CD68 on all CD14+ cells from these samples led us to conclude that these cells are macrophages. No natural killer or B cells and very few T cells (<0.05% of leukocytes and only in a minority of samples) were detected in the cervical brushes.

### 3.3 Prospective study

We compared data on the presence of cervical leukocytes in the late first and second trimester between the women who subsequently delivered at term (*n* = 76) and the women who experienced recurrent SPTB, either early (<34 weeks; *n* = 6) or late (34–36 + 6 weeks; *n* = 12). None of the participants had a late miscarriage. Fig. 2 contains representative flow cytometric plots of samples in which leukocytes were detected (Fig. 2A) or were undetectable (Fig. 2C). Fig. 2D plots for each participant the proportion of host-derived cells with a leukocyte phenotype (CD45+). Table 2 summarises the leukocyte subset detection rates for each participant group. Leukocytes were detected in 2 out of 6 (33%, 95% CI 10–70%) cytobrush samples from participants who delivered at <34 weeks compared with 7 out of 12 (58%, 95% CI 32–81%) of the late SPTB and 53 out of 76 (70%, 95% CI 60–80%) of the participants who delivered at term.

We then compared the distribution of cervical leukocyte subsets among outcome groups. None of the participants who gave birth before 34 weeks had any detectable macrophages (0%, 95% CI 0–39%; Fig. 2E and Table 2) compared with 7 out of 12 (58%, 95% CI 32–81%, *P *< 0.05) of the late SPTB and 51 out of 76 (67%, 95% CI 57–77%, *P *< 0.001) of the participants who delivered at term. Histological examination of the placenta and membranes was performed on all early SPTB (before 34 weeks); in 5 out of 6 cases there was evidence of chorioamnionitis.

Cervical leukocyte distribution was compared with cervical length and cervico-vaginal fetal fibronectin at 22–24 weeks. Whilst the number of participants who experienced a recurrence of SPTB was low, of the 6 participants who received ultrasound-indicated cerclage because of the early detection of a short cervix (<25 mm), 5 had >50 ng/mL fetal fibronectin at 22–24 weeks. Of these, 4 had no detectable cervical leukocytes. Of the 14 participants who received cerclage and gave birth at term, only 2 lacked cervical leukocytes and these were not the same 2 participants who had short cervices.

**Fig. 3:**
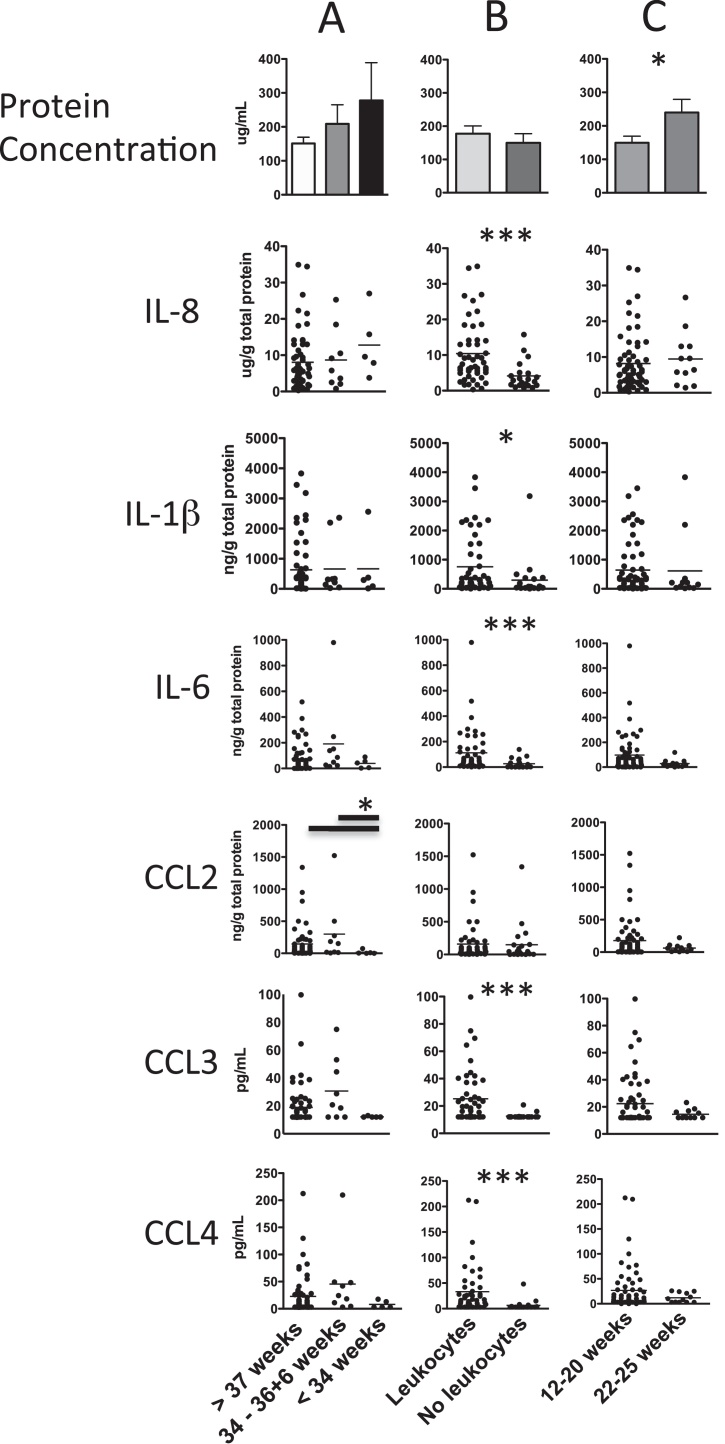
Differences in total protein, cytokine and chemokine levels in the cervical brush washes of different participant groups (*n* = 71). (A) Participants were grouped according to birth outcome. (B) Participants were grouped according to the presence or absence of cervical leukocytes. (C) Participants were grouped according to the stage of gestation when the sample was taken. Total protein is shown as mean with standard error. Cytokines and chemokines are shown as dots with horizontal lines indicating means. The limits of detection for CCL3 and CCL4 were 12.5 pg/mL and 3 pg/mL respectively. Samples with undetectable amounts of these cytokines were given the value of the detection limit. * = *P *< 0.05; ** = *P *< 0.01; *** = *P *< 0.001 between groups using the Mann–Whitney *U* test.

We measured IL-8, IL-6, IL1β, IL-10, IFNγ and TNFα in addition to the CC-type chemokines that attract CCR1- and CCR2-expressing cells (CCL2, CCL3 and CCL4) in cervical brush supernatants of 72 of the 94 participants in the study. IFNγ, TNFα and IL-10 were detected in less than 20% of samples and only in small amounts. Detection of these cytokines tended to occur in samples with a large amount of protein, the presence of T cells or high levels of other cytokines (data not shown). All of the proteins assayed with the exception of CCL2 had reduced levels in samples with no leukocytes, even though the total protein was equivalent between those with and without leukocytes (Fig. 3B). In contrast, CCL2 was reduced in the early preterm group compared with the late preterm and term groups (Fig. 3A). Thus, CCL2 above 75 ng/g and/or the presence of macrophages increased the specificity for birth after 34 weeks to 82% (95% CI 73–91%) compared with 66% (95% CI 57–75%) for macrophages alone. Even though protein content in the cervical washes increased with advancing gestation, the levels of measured cytokines and chemokines remained the same per gram of total protein. Of all factors measured, IL-6 had the strongest correlation with leukocyte proportions of the total host-derived cell content (*r*^2^ = 0.49, *P *< 0.001, data not shown).

The observed cervical immune phenotype of undetectable macrophages and low CCL2 was tested against the current factors used in early risk stratification (previous SPTB <34 weeks or cervical length <25 mm). We chose 18 weeks as the cut-off for the detection of a short cervix as we are aiming to develop an algorithm that could potentially inform decisions ahead of the optimal window for treatment with progesterone (Romero et al., 2014), cervical cerclage (Romero et al., 2006) and antibiotics (Joergensen et al., 2014). Cervical immune phenotype predicted SPTB before 34 weeks better than previous SPTB <34 weeks or short cervix because of its ability to rule out the large number of participants who had a history of SPTB <34 weeks, but who lacked the high-risk immune phenotype (Table 3). There was no difference between the two models for predicting overall preterm birth.

## 4 Discussion

This study provides a detailed assessment of cervical cellular immunity in pregnant women with a history of SPTB. The women destined to deliver before 34 weeks’ gestation had an absence of cervical macrophages and low levels of CCL2 in cervical mucus early in pregnancy. The paucity of these cervical antimicrobial components may have predisposed these women to ascending bacterial translocation and the chorioamnionitis that was apparent in the extraembryonic membranes in 5 out of 6 cases.

We found that the dominant population of cervical leukocytes are polymorphonuclear cells, probably neutrophils, with a minor population of macrophages. The finding that PMN and macrophages are the most prevalent cervical leukocytes is in agreement with a previous study in which immunohistochemistry was performed to identify and count cervical leukocytes (Whitworth et al., 2007). Whitworth et al. also reported low numbers of macrophages in cervical brush samples where the delivery was before 35 weeks. We rarely detected lymphocytes, although they have been reported in women attending a genitourinary infection clinic (Prakash et al., 2001) and in cervices of women with cervicitis, either clinically apparent or sub-clinical (Ibana et al., 2012, Ling et al., 2011). A systematic analysis of cervical leukocytes from non-pregnant women has not as yet been performed; however, it is interesting to note that routine microscopic analysis of Pap-stained cells from non-pregnant women reveals cells with tetralobular nuclei (neutrophils) intermingling with squamous epithelial cells. Only occasionally are cells with large vacuoles characteristic of macrophages, apparently suggesting that increased cervical macrophages might be a feature of pregnancy.

The only difference detected between the surface proteins on blood-borne monocytes and cervical macrophages was the expression of the adhesion molecules, CD103 (integrin αE) and CD68. As such it is tempting to assume that cervical leukocytes are derived predominantly from blood-borne precursors that exit the circulation and migrate through the epithelium. However, recent evidence suggests that tissue-resident myeloid cells such as Kupffer cells in the liver, Langerhans cells in the skin and microglia in the brain might be derived from progenitors that predate haematopoietic stem cells during embryonic development (Schulz et al., 2012). These long-term resident cells have been shown to respond to damage to their local environment and participate in its repair in response to inflammatory signals (Davies et al., 2013). As human tissue-resident myeloid cells are indistinguishable from blood monocyte-derived macrophages, it is not possible to ascertain the source of cervical macrophages at present. It is possible that both resident and blood-derived cells contribute to the cervical macrophage population through the continuous low-grade inflammatory environment maintained by low levels of cytokines and chemokines in the cervix.

In this report we have described a unique phenotype of a subset of women who are at a heightened risk of preterm birth. This phenotype is characterised by the absence of phagocytic leukocytes, particularly macrophages, and low levels of secreted CCL2. This subset overlaps with approximately 60% of the women who receive cerclage because they have a short cervix (<25 mm) before 18 weeks and/or have >50 ng/mL cervico-vaginal fetal fibronectin at 22–24 weeks. Importantly, this subset also includes women whose preterm delivery was not predicted by current clinical screening protocols. It does not include women who deliver at 34–36 + 6 weeks with no other predictors and average levels of cervical cytokines and chemokines. SPTB in this group remains unexplained. Only levels of CCL2 above 75 ng/g total protein added further specificity for birth after 34 weeks among women who lacked cervical macrophages. Both CCL2 and IL-8 are produced by cervical epithelial cells (Kleine-Lowinski et al., 2003) and both have been the target of disruption by invading pathogens at the genital and other mucosal sites (Darveau et al., 1998, Kleine-Lowinski et al., 2003). IL-6 levels had the strongest correlation with the leukocyte proportion of the host-derived cells, suggesting that IL-6 might be secreted from the leukocytes themselves. This correlation was surprising as IL-6 is highly variable among individuals in serum (Christ-Crain and Opal, 2010).

It is of interest that the high-risk immune phenotype was detected in 20% of participants who delivered at term. This highlights the fact that the anti-microbial barrier function of the cervical canal is likely to depend on a number of different but complementary factors (Cole, 2006, Mestecky et al., 2005). The control group (i.e. those delivering at term) in this study is not necessarily representative of the normal pregnant population as they were being seen in a specialised preterm birth clinic because of a history of SPTB. It is necessary to replicate this study in women with no identifiable risks.

In conclusion, we propose that bacterial invasion of the intrauterine space during pregnancy and the ensuing inflammatory response that occurs with the onset of preterm labour may be preceded by a period of inadequate cervical immune defence against ascending infection. The presence of phagocytic leukocytes at the distal cervix and endocervical canal and the chemokines that support their migration are features of pregnancies that are likely to go to term, and failure to detect even a small number of leukocytes by highly sensitive flow cytometric methods is a marker of heightened risk of preterm birth. Furthermore, this phenotype is evident as early as 12 weeks and offers significant improvement in current early risk stratification for SPTB, thus creating a gestational window of opportunity for interventions that could potentially prolong pregnancy. Our observations indicate that cervical leukocytes and particularly macrophages may play a critical role in cervical immunity and specifically in preventing microbial translocation to the feto-placental unit. It may therefore be possible to identify mothers at high risk of SPTB in which microbes are the probable trigger.

## Figures and Tables

**Table 1:** Summary of the clinical characteristics of participants.

	Term (>36 + 6 weeks)	Late preterm (34–36 + 6 weeks)	Early preterm (<34 weeks)
	(*n* = 76)	(*n* = 12)	(*n* = 6)
Age (years, mean ± SD)	32.5 ± 5.5	34.4 ± 5.1	32.5 ± 4.6
BMI at ∼10 weeks (mean ± SD)	26.4 ± 5.7	26.0 ± 4.8	26.2 ± 6.0
Smoked during pregnancy (*n*, %)	5, 6.6%	0, 0%	1, 17%

Ethnicity (*n*, %)			
White	46, 61%	7, 58%	5, 83%
Afro-Caribbean	17, 22%	2, 17%	1, 17%
Asian	13, 17%	3, 25%	0, 0%

History (n, %)			
Previous SPTB <34 weeks^a^	36, 47%	3, 25%	4, 67%
Previous SPTB ≥34 weeks^b^	9, 12%	7, 58%	1, 17%
Previous LMC	31, 41%	2, 17%	1, 17%

Sample donated			
12–20 weeks (n, %)	66, 87%	9, 75%	5, 83%
Progesterone	0, 0%	0, 0%	2, 33%
Ultrasound indicated cerclage			
(Cervix <25 mm; n, %)	2, 2.6%	5, 42%	1, 17%
Elective cerclage (n, %)	12, 16%	0, 0%	3, 50%
Cervico-vaginal fetal fibronectin			
>50 ng/mL detected at 22–24			
weeks (n, %)	6, 7.9%	5, 42%	3, 50%
Shortest cervical length (mm, mean ± SD)	29.5 ± 4.4	24.8 ± 6.8	26.5 ± 8.0
Positive result in infection screen			
(*n*, %)^c^	29, 38%	2, 17%	1, 17%

SPTB spontaneous preterm birth, LMC late miscarriage (16–23 + 6 weeks).

a One or more SPTB <34 weeks. These women may also have had LMC or SPTB ≥34 weeks.

b One or more SPTB ≥34 weeks. These women may also have had LMC.

c Infections screened for bacterial vaginosis, *Trichomonas vaginalis*, gonococcus, chlamydia, group B *Streptococcus*, candidiasis and asymptomatic bacteriuria.

**Table 2:** Summary of the association of detected leukocyte subsets and CCL2 with SPTB.

	Term (>36 + 6 weeks)	Late preterm (34–36 + 6 weeks)	Early preterm (<34 weeks)
PMN (CD45+CD13+ CD16+CD66b+)	53/76 (70%)	7/12 (58%)	2/6 (33%)
Macrophages (CD45+ CD13+CD14+ CD68+HLADR+)	51/76 (67%)	7/12 (58%)	0/6 (0%)
CCL2 (>75 ng/g total protein)	26/58 (45%)	5/9 (56%)	0/5 (0%)
Macrophages or CCL2 (>75 ng/g total protein)	46/58 (79%)	9/9 (100%)	0/5 (0%)

PMN: Polymorphonuclear cells.

**Table 3:** Comparison of likelihood ratios (LR+) for predicting SPTB based on history and cervical length versus cervical immune phenotype.

	LR+ for SPTB <37 weeks	LR+ for SPTB + <34 weeks
(1) Previous SPTB <34 or cervical length <25 mm before 18 weeks	1.1 (95% CI 0.71–1.8)	1.7 (95% CI 1.1–2.6)
(2) Undetectable cervical macrophages and CCL2 <75 ng/g	1.7 (95% CI 0.73–4.1)	5.6 (95% CI 3.3–9.3)*
Both 1 and 2	4.1 (95% CI 1.4–12.4)	9.2 (95% CI 3.8–22)*

* *P* < 0.05 in comparison with (1).
