# Supplementary material for: Cervical leukocytes and spontaneous preterm birth
Source: J Reprod Immunol. 2016 Feb;113:42–9. doi: 10.1016/j.jri.2015.11.002 (PMC4764650; doi:10.1016/j.jri.2015.11.002)
Supplement: Supplementary file 1 [file mmc1.docx]

Supplementary Table 1. Summary of the clinical characteristics of participants whose samples were excluded owing to blood contamination of the sample

Term (>36+6 weeks) Late Preterm (34–36+6 weeks) Early Preterm (<34 weeks)

(n=9) (n=3) (n=2)

Age at entry (years, mean ± SD) 30.3 ± 5.0 31.7 ± 7.1 34.5 ± 7.8

BMI at ~10 weeks (mean ± SD) 27.4 ± 8.0 25.2 ± 6.0 25.8 ± 1.0

Smoked during pregnancy (n, %) 0, 0% 0, 0% 1, 50%

Ethnicity (n, %)

White 4, 44% 2, 67% 0, 0%

Afro-Caribbean 2, 22% 1, 33% 1, 50%

Asian 3, 33% 0, 0% 1, 50%

Supplementary Table 2. Summary of the clinical characteristics of participants whose samples were excluded because of spoilage or insufficient cell numbers

Term (>36+6 weeks) Late Preterm (34–36+6 weeks) Early Preterm (<34 weeks)

(n=7) (n=0) (n=0)

Age at entry (years, mean ± SD) 36.6 ± 6.7

BMI at ~10 weeks (mean ± SD) 25.4 ± 6.1

Smoked during pregnancy (n, %) 1, 14%

Ethnicity (n, %)

White 2, 29%

Afro-Caribbean 3, 43%

Asian 2, 29%
